# Targeted genome engineering in *Caenorhabditis elegans*

**DOI:** 10.1186/s13578-016-0125-3

**Published:** 2016-12-08

**Authors:** Xiangyang Chen, Xuezhu Feng, Shouhong Guang

**Affiliations:** School of Life Sciences, CAS Center for Excellence in Molecular Cell Science, University of Science and Technology of China, Hefei, Anhui 230027 People’s Republic of China

**Keywords:** Genome engineering, Cre/LoxP, FLP/FRT, MosTIC, ZFNs, TALENs, CRISPR/Cas9

## Abstract

The generation of mutants and transgenes are indispensible for biomedical research. In the nematode *Caenorhabditis elegans*, a series of methods have been developed to introduce genome modifications, including random mutagenesis by chemical reagents, ionizing radiation and transposon insertion. In addition, foreign DNA can be integrated into the genome through microparticle bombardment approach or by irradiation of animals carrying microinjected extrachromosomal arrays. Recent research has revolutionized the genome engineering technologies by using customized DNA nucleases to manipulate particular genes and genomic sequences. Many streamlined editing strategies are developed to simplify the experimental procedure and minimize the cost. In this review, we will summarize the recent progress of the site-specific genome editing methods in *C. elegans*, including the Cre/LoxP, FLP/FRT, MosTIC system, zinc-finger nucleases (ZFNs), transcriptional activator-like nucleases (TALENs), and the clustered regularly interspaced short palindromic repeats (CRISPR)/Cas9 nuclease. Particularly, the recent studies of CRISPR/Cas9-mediated genome editing method in *C. elegans* will be emphatically discussed.

## Background

The nematode *Caenorhabditis elegans* is a model organism, which is widely used in genetic and biomedical research [1]. The *C. elegans* genome consists of approximately 97 mega base pairs and encodes roughly twenty thousand protein-coding genes (WormBase referential freeze WS254, May 2016), yet more than 40% of its genes have considerable homologies in other organisms [2]. In addition, a lot of genetically characterized nematode strains, including mutations and transgene integrations, can be easily obtained from the Caenorhabditis Genetics Center (CGC), which considerably aid world-wide researchers [3].

Many genome engineering methods have been developed to induce random mutations and chromosomal structure alterations. Chemical mutagens, including ethyl methane sulfonate (EMS), *N*-ethyl-*N*-nitrosourea (ENU) and ultraviolet-activated trimethylpsoralen (UV/TMP) [4, 5], are widely used to induce DNA lesions in forward genetic screens. Ionizing and UV radiation mutagenesis are typically employed to generate large sequence deletions or chromosomal rearrangements, such as chromosomal duplications, inversions and translocations. Transposon-mediated insertional mutagenesis approaches, including Tc1 and Mos1 system, have been applied in genetic screens and significantly eased the identification of the putative mutations through an optimized inverse PCR approach [4, 6]. Recently, an optogenetic mutagenesis method was developed by adopting the mini Singlet Oxygen Generator (miniSOG) system, which expands the toolbox for forward genetic screening [7].

Exogenous DNA fragments can be inserted into the *C. elegans* genome. After microinjection of a DNA plasmids mix solution into the gonad, the plasmids DNA undergoes intermolecular ligation and rearrangement to form multi-copy extrachromosomal DNA arrays which are semistable and inherited to a part of the progenies [8]. Thereafter, the extrachromosomal arrays can be stably integrated into the genome by mutagens or radiations to generate high copy numbers of transgenes [9]. In addition, microparticle bombardment method was successfully applied to directly produce low-copy integrated transgenic lines in *C. elegans* [10]. High copy integrated arrays are prone to be silenced, yet low-copy transgenes permit relatively stable expression of proteins of interest in the *C. elegans* germline. Recently, Frøkjær-Jensen et al. developed a miniMos strategy to arbitrarily insert single copy exogenous DNA fragments into the genome [11]. The miniMos is a truncated Mos1 transposon that is loaded with large DNA fragments and insert into the chromosome at high frequency through co-injection of plasmid expressing the Mos1 transposase. A limited number of *C. elegans* strains carrying the miniMos1 site have been created to facilitate this single-copy transgene integration.

Although the genome-wide mutagenesis methods are broadly used to create nematode strains with mutations or integrated transgenes, sequence-specific gene editing are hardly achieved through these approaches. Great efforts have been devoted to develop a series of targeted genome editing technologies in *C. elegans*, including Cre/LoxP and FLP/FRT recombination, Mos1 excision-induced transgene-instructed gene conversion (MosTIC), zinc-finger nucleases (ZFNs), transcription activator-like effector nucleases (TALENs) and the recent developed clustered regularly interspaced short palindromic repeats (CRISPR) RNA-guided Cas9 nuclease technology. These methods, especially the CRISPR/Cas9 technology, remarkably assist the generation of nematode strains with desired sequence alterations on genes of interest.

In this review, we will summarize these site-specific genome engineering technologies and discuss the optimization of these methods to provide appropriate genome editing strategies for different purposes.

### *FLP/FRT* and *Cre/LoxP* recombination technologies

*FLP/FRT* and *Cre/LoxP* systems are broadly applied to modify genome to induce chromosomal rearrangements [12, 13] and conditionally activate or inactivate gene expression [14]. The site-specific recombinases *Flp* and *Cre* recognize the *FRT* (for short flippase recognition target) and *LoxP* (for locus of X-over P1) sites, respectively. Then *Flp* and *Cre* catalyze the recombination of two *FRT* sites and *LoxP* sites, to induce excision or inversion of the flanked DNA segment, depending on the orientations of the two repeat elements. These recombination-mediated editing methods have been successfully applied to induce sequence deletions or inversions in vivo to control gene expression in *C. elegans* [14]. Typically, repeats with the same orientation result in an excision of the contained DNA segment, while inverted repeats lead to inversion of the DNA sequences between the two repeat elements. Thus, a particular gene can be activated by inverting an originally inverted promoter or coding sequence, or by eliminating a DNA fragment containing a transcriptional stop. Similarly, genes can be inactivated through the removal of the promoter or coding regions. Conditional gene manipulation is achieved via the spatial or temporal expression of recombinases, driven by tissue specific promoters or a heat shock promoter. In addition, the FLP/FRT and Cre/LoxP approaches are used in conjugation with the single-copy transgenic technologies to facilitate the removal of co-integrated positive selection markers, which are flanked by *LoxP* or *FRT* sites, and streamline the construction of transgenic animals [15–19].

### MosTIC-induced targeted gene conversion

Mos1 transposon, a member of the mariner/Tc1 family, was originally identified in the fruit fly *Drosophila mauritiana*. Mos1 transposon can randomly insert into the *C. elegans* genome by Mos1 transposase-mediated cleavage and integration processes [20, 21]. Thereafter, for animals with integrated Mos1 transposons at particular genome locations, the transient expression of Mos1 transposase through extrachromosomal array under the heat-shock promoter *Phsp*-*16.48* or germline specific promoter *Pglh*-*2* can induce chromosomal breaks at the Mos1 site on the genome [22, 23]. These double-strand breaks (DSB) are further repaired, in the presence of donor repair templates, to elicit precise sequence alterations, including point mutations, deletions and insertions [23, 24]. The Mos1 excision-induced transgene-instructed gene conversion (MosTIC) system, like the *Cre/LoxP* and *FLP/FRT* method, relies on the prior presence of animals containing Mos1 insertion sites at the destined genomic locus. A library of nematode strains with Mos1 insertion sites have been generated by the NemaGENETAG consortium for the *C. elegans* community [25, 26].

A Mos1-mediated single-copy insertion (MosSCI) system has been developed to construct single copy transgenic lines [27]. A number of *C. elegans* strains were engineered with *Mos1* elements inserted at certain intergenic genomic loci for the routine insertion of transgenes. The single copy transgenes likely express the recombinant proteins at normal physiological levels and escape the small RNA-mediated transgene silencing in the germline. Therefore, the MosSCI method provides a platform to investigate the genes involved in germline development and progeny propagation.

### ZFNs and TALENs create DNA lesions through the utility of sequence-specific DNA-binding modules

Site-specific nucleases mediated targeted genome engineering has been demonstrated to be a wide applicable solution for effective genome manipulation in a variety of organisms. These engineered nucleases cleave specific genomic locus via the use of custom-designed DNA binding domains that recognize destined DNA sequences. Two of methods, ZFNs and TALENs, have been well developed and applied to genome editing in many organisms, including *C. elegans*.

**Fig. 1:**
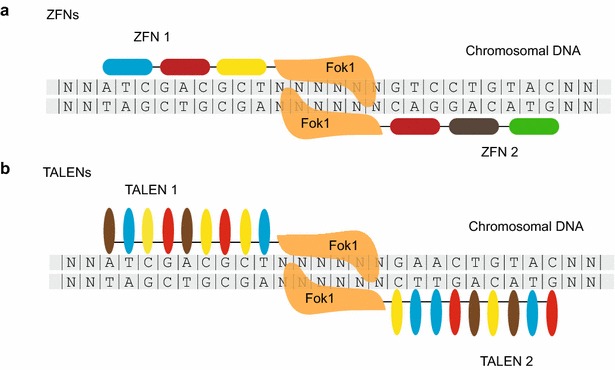
Schematic representation of the ZFN and TALEN pairs. **a** Illustration of a pair of ZFNs bound to specific DNA sequences. Zinc finger modules are shown as *rounded rectangle boxes*. Each zinc finger binds to a particular nucleotide triplet. **b** Illustration of a pair of TALENs. TALE modules are represented as *ellipses* and each recognizes a specific nucleotide. The DNA binding domains of ZFN and TALEN are fused to a cleavage domain of FokI nuclease, therefore guide the FokI nuclease to the desired genomic loci and cleave the flanked spacer sequences

ZFNs are artificially engineered proteins generated by fusing tandem Cys2His2 zinc finger domains with a DNA cleavage domain from the restriction endonuclease FokI [28]. One zinc finger module recognizes a particular 3-bp DNA sequence, thus three zinc finger modules are typically linked together to confer a zinc finger protein (ZFP) that binds 9-bp DNA sequences (Fig. 1a). *Fok*I nuclease cleaves DNA adjacent to the binding site to elicit a DSB. Since the catalytic domain of *Fok*I must dimerize to be active, two ZFN nucleases are usually engaged at the same time, which allows a combined recognition sequence of 18-bp. More recent studies use ZFNs with 4, 5 or 6 zinc fingers to specify longer and rarer cleavage targets, yielding less off-target activity. The DSBs were then repaired through non-homologous repair processes that further introduce mutations at the cleavage site. Theoretically, ZFNs can be designed to cleave DNA at any genomic loci by combining distinct zinc finger modules with different specificities. Morton et al. reported that ZFNs can induce targeted DSBs on extrachromosomal and chromosomal targets in nematode somatic cells at high frequency [29]. Wood et al. utilized this method to modify genes in the germline to generate heritable mutations of selected genes, including integrated exogenous *gfp* sequence and endogenous genes, *ben*-*1* and *rex*-*1* [30].

TALENs function like ZFNs, except that the non-specific FokI nuclease is fused to tandem transcription activator-like (TAL) domains. TAL effectors (TALEs) are proteins produced by Xanthomonas bacteria when they infect plants [31]. In host plant cells, TALEs bind specific promoter sequences and regulate gene expression to assist infection. Typically, TALEs bind target DNA sequence via tandem repeats of 34 amino acids monomers. Each repeat is highly conserved and differs only by two variable amino acids at positions 12 and 13, which are called the repeat variable di-residues (RVDs) that determine the DNA-binding specificity [32, 33]. Unlike zinc fingers domain, each TAL domain just binds to a single nucleotide, rather than three nucleotides (Fig. 1b). The serially combined TAL domains direct the FokI nucleases to desired genomic DNA sequences and generate DNA lesions. TALENs has been successfully applied to induce heritable genome editing of a series of *C. elegans* genes [16, 30]. Precise knockins via homology directed repair (HDR) has also be accomplished through the TALENs technology by exogenously supplying single strand DNA oligonucleotides (ssODNs) as a repair template for HDR [16]. Recently, Cheng et al. developed a strategy to generate inducible gene knockouts via temporal or spatial expression of somatic TALENs [34].

### The CRISPR/Cas9 system is directed by small guide RNA to cleave targeted DNA sequences

Although ZFNs and TALENs provide a platform for targeted genome editing efficiently with less sequence requirements than the MosTIC system, the processes to design and build the sequence specific nucleases are time-consuming, cumbersome and expensive, which hinder their applications. Recently, the clustered regularly interspaced short palindromic repeats (CRISPR) RNA-guided Cas9 nucleases has revolutionized the genome engineering technologies in numerous organisms [35]. The CRISPR/Cas9 technology is a versatile RNA-directed genome editing method that uses small guide RNA to recognize complementary DNA sequences, direct Cas9 nucleases to the targeted site, and elicit DSBs.

The CRISPR/Cas system is a heritable and adaptive immune system presenting in bacteria and archaea, which confers resistance to foreign genetic elements that are embedded within plasmids or phages [36]. Clustered regularly interspaced short palindromic repeats (CRISPR) are segments of repeated sequences separated by unique short spacer DNA elements originated from the DNA of a previously exposed bacteriophage or plasmids. Generally, a long precursor CRISPR RNA (pre-crRNA) is transcribed from the CRISPR region and subsequently processed by Cas nucleases and accessory factors to form a mature crRNA. Through the combined action of crRNA and Cas proteins, the targeted DNA sequence can be recognized and cleaved to defense the infection of invaded nucleic acids.

**Fig. 2:**
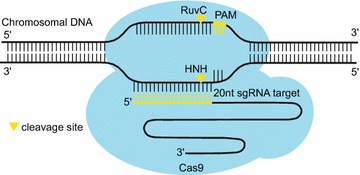
Schematic of the CRISPR/Cas9 system. Cas9 nuclease is directed by small guide (sg)RNA to cleave the desired DNA sequences. The first 20-nt of the sgRNA recognize its targeted DNA through base-paring interaction. A PAM motif on the DNA target is required for the enzymatic activity of Cas9 protein. The RuvC and HNH endonuclease domains of Cas9 cleaves one strand of DNA, respectively, to generate a double-stranded DNA break approximately 3 bp upstream of the PAM

Among diverse Cas proteins, the Cas9 nuclease from the type II CRISPR system, has been most widely used for genome editing in a series of organisms. Cas9 is directed to DNA sequences by a duplex of two RNAs: the crRNA that contains a 20-nt guide sequence recognizes the targeted DNA and the supporting trans-activating crRNA (tracrRNA) that hybridizes with the crRNA and binds to the Cas9 protein [37]. Current CRISPR system fuses the crRNA–tracrRNA duplex into a chimeric single guide RNA (sgRNA) [35] (Fig. 2). The 20-nt guide sequence located at the 5′-end of the sgRNA complements to the targeted DNA sequence via Watson–Crick base-pairing. A protospacer adjacent motif (PAM) immediately downstream the targeted site in the DNA sequence is required for the cleavage reaction by Cas9 nuclease, which further increases the specificity of target recognition. The Cas9 nuclease can be guided to cleave any desired genomic sequence that contains a GG dinucleotide, which provide a high versatility to choose the targeted editing locus. DSBs are then generated by Cas9 nuclease. As a consequence of non-homologous end joining (NHEJ) repair, mutations will be introduced at the desired site.

The CRISPR/Cas9 technology has been successfully applied to induce heritable gene alterations in *C. elegans* [15, 16, 38–44]. A number of methods to deliver the Cas9 protein and sgRNA to the germline have been developed. Plasmids that express Cas9 protein under the *eft*-*3* promoter or *hsp*-*16.48* heat-shock promoter and sgRNA under a *U6* promoter are constructed to drive the expression of Cas9 and sgRNA in the germline after a microinjection into the gonad [39, 41, 44]. Additionally, in vitro transcribed sgRNA and *Cas9* mRNA or purified Cas9 protein can also be introduced into the gonad by microinjection [16, 40, 42, 43]. Interestingly, Liu et al. developed a CRISPR-Cas9 feeding system that feeds the *Ppie*-*1::Cas9* transgenic animals with bacteria expressing sgRNA, which may facilitate high throughput genetics screening [45].

In addition, conditional gene knockout can be achieved by using a somatic expressed Cas9 protein under a tissue specific promoter or a heat-shock promoter [45–47]. Therefore, DNA indels are generated at predetermined developmental stages in specified somatic tissues of *C. elegans,* which produce tissue specific loss-of-function phenotypes.

Besides imprecise genome editing via non-homologous end joining and DNA repair, the CRISPR/Cas9 system can precisely modify a target sequence through HDR under the guidance of exogenously supplied DNA templates [15, 39, 40, 44, 48–51]. A number of repair templates have been used in *C. elegans*, including short ssODNs and long double-strand DNA, such as plasmids and PCR-generated DNA fragments. The plasmid DNA templates usually carry 300–2000 bp of homologous sequence arms flanking the desired modifications [15, 40, 44]. Point mutations and large DNA fragments insertions can be introduced into the genome through the use of plasmids. Interestingly, Paix et al. reported a convenient method by using a linear PCR fragment with short homology arms, which bypasses the plasmid construction process [49, 52]. The optimal length of the homology arms was estimated to be roughly 30- to 60-bp. Other groups also used a short ssODNs to direct precise editing [48–51, 53, 54]. ssODNs can be chemical synthesized and directly microinjected into the gonad without amplification or cloning procedures. Usually, a donor ssODN contains the desired nucleotide variation(s) flanked by 30-80 nucleotides on both sides that match the targeted sequence.

In addition to editing a single gene, the CRISPR/Cas9 technology has been applied to manipulate chromosomes and elicit chromosomal rearrangements [55–57]. A number of DSBs can be introduced in the presence of multiple sgRNAs simultaneously. Thereafter, large genomic fragments can be reversed, deleted, or translocated to other chromosomal loci. For example, our lab has reported the use of dual sgRNA strategy to direct reciprocal chromosomal translocations in *C. elegans* [58]. The nematode strains with specific chromosomal rearrangements can serve as genetic balancers for the screening and maintenance of essential genes [59].

Recent progress has developed the CRISPR interference (CRISPRi) and CRISPR-on strategies to regulate gene transcription in *C. elegans* [60]. A catalytically inactive form of Cas9, dCas9, was fused with transcription activator or repressor to modulate gene expression at or near their endogenous expression location(s) through target-specific gRNAs (ts-gRNAs). In addition, a DNA methyltransferase can be fused to dCas9 to sequence specially methylate genome DNA in mammalian cell lines [61].

#### Optimization of sgRNA and Cas9 protein

Although a series of editing experiments have been performed with the use of many different sgRNAs and various delivery strategies, there is still a lack of systematic prediction of the cleavage efficiency of a particular sgRNA. It is pivotal to develop strategies to design sgRNAs with higher efficiency. The combination of multiple sgRNAs targeting the same gene was shown to improve cleavage efficiency [49, 58, 62]. Farboud and Meyer reported that guide RNAs with a GG motif at the 3′ end of their target sequences can dramatically improve the editing efficiency [63]. A modified sgRNA (F + E) with an extended Cas9 binding structure yet lack of a putative Pol III terminator increased activity in both mammalian cells and *C. elegans* [54, 64].

The requirement for a PAM motif in the targeted DNA limits the choice of sgRNA sequences. To overcome this constraint, modified Cas9 nucleases with altered PAM specificities have been developed that expand the target repertoire and ease the high-throughput fluorescent protein tagging of endogenous genes [65]. For example, Bell et al. have successfully applied two modified *Cas9* proteins that recognize NGA and NGCG PAM respectively to *C. elegans* [66].

#### Efficient identification of genome-modified *C. elegans* strains

Animals with targeted gene modifications can be obtained through phenotypic analysis and PCR screening. Animals with morphological changes, such as Dpy, Unc and Rol, can be easily visualized and selected. Integrated transgenic lines tagged with fluorescent proteins can be identified under a fluorescence microscope. For genes of which loss-of-function result in no obvious phenotypes, the mutations can be detected via PCR screening. The PCR amplicons surrounding the sgRNA sites can be analyzed using T7 endonuclease I (T7E1) or restrictive endonuclease digestion. Moreover, simultaneous introduction of multiple sgRNAs lead to the removal of large DNA chunks between the sgRNAs, which simplifies the identification of deletion mutants by PCR amplification followed by agarose gel electrophoresis [58, 62]. The integrated transgenes can be identified by PCR amplified with appropriate primers as well.

**Fig. 3:**
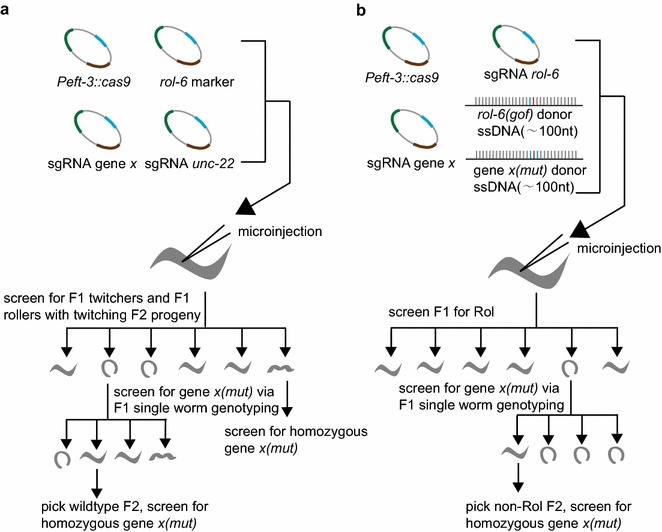
The co-CRISPR and co-conversion strategies for the detection of targeted genome modifications. **a** The co-CRISPR strategy used *rol*-*6(su1006)* expression plasmid as a co-injection marker and an *unc*-*22* sgRNA as a co-editing marker. F1 animals with both twitching and rolling phenotypes are selected. The twitching F2 animals are further screened by single worm PCR to identify the animals with gene *X* mutation. **b** The co-conversion strategy used a donor oligonucleotides carrying the *rol*-*6(su1006)* mutation as both co-injection and editing marker. F1 roller animals are screened by single worm PCR to identify the animals with gene *X* mutation. The figure was adapted from Arribere et al. [51]

Several screen methods have been developed to assist the identification of genome editing events in *C. elegans*. Kim et al. used a co-CRISPR strategy with two sgRNAs to simultaneously edit the genome [50], of which one acts as a co-sgRNA to induce an easily recognizable phenotype and the other sgRNA targets the gene of interest (Fig. 3a). The visible phenotype generated by the co-sgRNA allows to identify animals in which Cas9 is active to edit genomic DNA. The co-CRISPR strategy dramatically increased the frequency of detecting NHEJ or HDR events targeting specified genes. Arribere et al. further optimized this co-CRISPR method and devised a co-conversion strategy to detect gene editing events via the application of several gain-of-function alleles [51], in which a donor template was co-injected simultaneously to create a dominant marker mutation (Fig. 3b). The co-conversion strategy provides a platform for efficient marker-free recovery of HR directed precise genetic modifications. Ward then used a temperature-sensitive lethal mutation of the *pha*-*1* gene as a co-conversion marker and deactivated the NHEJ repair pathway via *cku*-*80* RNAi during the co-conversion procedure [54]. Animals rescued the *pha*-*1(e2123)* mutation were then selected and genotyped.

Selectable markers, including phenotypic selection markers and antibiotic-resistance markers, are inserted into the genome together with the desired genome alterations, to assist the detection of HDR events and the identification of integrated transgenic animals. *Cbr*-*unc*-*119*(+), a marker commonly employed in the MosSCI-mediated genome editing technology, has been applied to isolate CRISPR/Cas9-induced insertions by rescuing of a visible Unc phenotype [15]. Antibiotic-resistance markers, such as resistance against *blasticidin*, *hygromycin* and *neomycin*, are also used for mutants selection [18, 44, 50]. Additionally, the benomyl resistance provides an alternative counter selection strategy for targeted knock-in of specific DNA fragments at the *ben*-*1* locus [44]. While wild-type animals exhibit a visible paralysis phenotype when exposed to benomyl at 25 °C, the loss of function of *ben*-*1* by targeted transgene insertion confers benomyl resistance [58].

Several selection cassettes, each containing a visible phenotypic marker and an antibiotic-resistance marker, have been created to construct versatile plasmid vectors in conjunction with other functional DNA elements, which act as templates for homologous repair in *C. elegans*. For example, Norris et al. devised a dual-marker selection system that using a repair cassettes containing an antibiotic resistance gene (*Prps*-*27::neoR*) and a fluorescent visual marker (*Pmyo*-*2::gfp*) [17]. The antibiotic marker is used to detect worms carrying the repair templates and the fluorescent marker enables convenient selection of homologous recombinants. The cassette, flanked by *LoxP* sites, is inserted into an intron of *gfp* sequence and can be easily removed from the recombinant genome by injecting the plasmid expressing Cre recombinase in the germline. Dickinson et al. developed a self-excising cassette (SEC) for rapid identification of fluorescent protein knock-ins events [18]. The SEC is composed of three components: a drug-resistance gene (*hygR*), a visible phenotypic marker [*sqt*-*1*(*e1350*)], and a heat-inducible Cre recombinase. The SEC is flanked by *LoxP* sequences and can be easily excised from the genome after a heat shock treatment. These methods greatly minimized the time and labor requirements to identify precise genome modifications, enabled robust selection without large-scale PCR screening, and provided a streamlined platform for genome-wide fluorescent protein knock-ins.

#### High-throughput genome editing by CRISPR/Cas9 technology

The direct application of in vitro synthesized sgRNA and purified Cas9 protein have greatly eased the genome editing experiments, yet recent efforts are devoted to simplify the construction of vectors expressing sgRNA and plasmids containing homologous repair templates. These methods streamlined the procedure for high-throughput genome editing by the CRISPR/Cas9 technology.

Ward utilized fusion PCR approach to generate linear DNA fragments to express sgRNA, bypassing the molecular clone steps [54]. Schwartz and Jorgensen have designed a convenient modular plasmid assembly strategy with high efficiency, termed as SapTrap [19]. In this method, all target specific DNA fragments, including guide RNA and short homology arms, are provided as annealed synthetic oligonucleotides. Other invariant modular components, including tag and marker cassettes and connector modules (CNCTR) are derived from the donor plasmids, which are digested by the restriction enzyme *Sap*I. These components are ligated in a fixed order to produce the targeting vector, using the Golden Gate assembly method. Moreover, the authors have generated a SapTrap donor plasmids library that supplies a variety of tags and connectors, allowing flexible tagging at specified genomic locus.

Paix et al. developed an in vivo recombination strategy to induce gene conversions in *C. elegans* [67]. This method combined short ssODNs and PCR fragments to introduce desired DNA sequences into specific genomic loci. The overlapping ssODNs initiate DNA repair in vivo and, assemble with each other to form an entire fragment, and are effectively inserted into the genome. ssODNs bridge multiple PCR fragments to chromosomal breaks, and induce an efficient insertions of the PCR fragments to defined genomic loci. This method eliminates the cumbersome and time-consuming molecular cloning procedures.

## Conclusions and perspectives

Genome engineering methods have marvelously promoted the forward and reverse genetic studies in *C. elegans*. Genome wide random mutagenesis can be conducted with diverse strategies, including chemical reagents, high-energy radiation and transposon insertions. Targeted genome editing technologies, which use site specific DNA nucleases to induce genome modifications, have tremendously simplified the manipulation of a selected DNA sequence in vivo. By combining both forward and reverse genetics, the function and mechanism of genes and biological processes can therefore be thoroughly investigated.

Many mutants, especially mutants with missense point mutations, exhibit no obvious phenotypes in various species including *C. elegans*. The reason could be the lack of observable phenotypes that the researchers investigated or gene compensations. In addition, many genes show synthetic phenotypes or only reveal noticeable defects under stress conditions. Therefore, null or multiple independent alleles are usually required to pinpoint the function of genes.

Distinct site-specific genome engineering technologies can be used according to the particular editing aims. The recombinases used in the Cre/LoxP, FLP/FRT systems and the mos1 transposase utilized in the Mos1 systems exclusively recognize specified DNA sequences, and therefore require for particular *C. elegans* strains carrying these sequence elements. On the other hand, ZFNs, TALENs and CRISPR can be engineered to recognize arbitrary DNA sequences in the genome and induce editing events independent of the prior existence of certain sequence elements. The pros and cons of different genome engineering tools and their applications in worm study are summarized in Table 1. Although the recent development of CRISPR/Cas9 technology has greatly simplified the gene manipulation processes with higher efficiency and wider applications, the non-CRISPR/Cas9 techniques can be used in combination with the Cas9 system to establish streamlined genome editing procedure. For example, researchers have combined Cre/LoxP and FRT/FLP recombination systems with CRISPR/Cas9 technology to conduct genome engineering experiments to acquire tagged animals.

**Table 1:** The pros and cons of different genome editing technologies in *C. elegans*

	Cre/loxP and FLP/FRT	MosTIC	ZFN	TALEN	CRISPR
Functional enzyme	Cre recombinase and FLP flippase	Mos1 transposase	Customized fokI	Customized fokI	Cas9
Recognition	Protein-DNA	Protein-DNA	Protein-DNA	Protein-DNA	RNA-DNA
Mechanism of action	DNA recombination	Mos1 transposon elimination	Induce DSBs	Induce DSBs	Induce DSBs
Sequence limit	LoxP or FRT sequence	Mos1 transposon	No	No	PAM motif
Genetic background	Strain with extrachromosomal array	Mos1 insertion strain	Any	Any	Any
Specificity design	Targeted sequence flanked by LoxP or FRT sites	Targeted modifications within repair templates	Zinc finger modules, each binds to a particular nucleotide triplet	TALE modules with each binds to a single nucleotide	The first 20-nt of the sgRNA
Application	Conditionally regulate gene expression, remove co-integrated selection markers	Precise sequence alterations	Gene KO	Gene KO, conditional gene KO and precise sequence alterations	Gene KO, conditional gene KO, precise sequence alterations and chromosomal engineering

Many genes play pleiotropic roles in various tissues or at different developmental stages. The conditional genome editing methods greatly facilitated the manipulation of these genes, by controllable gene activation or inactivation. The loss-of-function mutation of essential genes can be easily generated and maintained through the combination of CRISPR/Cas9 technology and the balancer system. High throughout genome editing, especially genome-wide fluorescent protein tagging, should be of much significance for the *C. elegans* community. In *C. elegans*, several groups have previously undertaken genome-wide expression projects by using extrachromosomal promoter::GFP or promoter::CDS::GFP reporters. Yet tagging fluorescence proteins into endogenous genes using the CRISPR/Cas9 technique will represent the native expression patterns and regulations. The recent developed editing strategies based on the optimization of the repair templates construction and the simplified screening methods for modified animals, provide the possibility to obtain a library of nematode strains with mutation or tagging of every single gene. Further optimization of the experimental operations will smooth the creation of these libraries to accelerate the research of *C. elegans* biology.

## Abbreviations

EMS: ethyl methane sulfonate
ENU: *N*-ethyl-*N*-nitrosourea
UV: ultraviolet
TMP: trimethylpsoralen
miniSOG: mini singlet oxygen generator
Lox P: locus of X-over P1
FLP: flippase
FRT: flippase recognition target
MosTIC: Mos1 excision-induced transgene-instructed gene conversion
ZFNs: zinc finger nucleases
TALEs: TAL effectors
TALENs: transcription activator-like effector nucleases
TAL: tandem transcription activator-like
CRISPR: clustered regularly interspaced short palindromic repeats
Cas: CRISPR-associated proteins
Cas9: native Cas9 nuclease
crRNA: CRISPR RNA
pre-crRNA: precursor CRISPR RNA
tracrRNA: trans-activating crRNA
sgRNA: single guide RNA
PAM: protospacer adjacent motif
DSB: double strand break
NemaGENETAG: nematode gene-tagging tools and resources
RVDs: repeat variable di-residues
HDR: homology directed repair
NHEJ: non-homologous end joining
ssODNs: single strand oligodeoxynucleotides
T7E1: T7 endonuclease I
RNAi: RNA interference
Dpy: dumpy
Unc: uncoordinated
Rol: roller
Cbr-unc-119(+): caenorhabditis briggsae unc-119(+)
PCR: polymerase chain reaction
neoR: neomycin-resistance gene
hygR: hygromycin-resistance gene
CNCTR: connector modules
ts-gRNAs: target-specific guide RNAs
dCas9: deactivated Cas9 nuclease
KO: knockout
